# Analysis of Himalayan marmot distribution and plague risk in Qinghai province of China using the “3S” technology

**DOI:** 10.1038/s41598-023-28414-5

**Published:** 2023-02-02

**Authors:** Hailian Wu, Haisheng Wu, Yongshun Wang, Hongying Li, Fuzhang Tian, Kuizhang Zhou, Zhizhen Qi, Yiquan Zhang, Qingwen Zhang, Xuefei Zhang

**Affiliations:** 1 Qinghai Institute for Endemic Disease Prevention and Control, Xining, 811602 Qinghai China; 2 Key Laboratory for Plague Control and Research of National Health Commission, Xining, 811602 Qinghai China; 3 Key Laboratory for Plague Prevention and Control of Qinghai Province, Xining, 811602 Qinghai China; 4 Qinghai Provincial Basic Geographic Information Center, Xining, 811602 Qinghai China; 5 Department of Clinical Laboratory, Affiliated Nantong Hospital 3 of Nantong University, Nantong, 226006 Jiangsu China

**Keywords:** Animal migration, Ecological epidemiology, Grassland ecology, Microbial ecology

## Abstract

To provide guidance for plague surveillance and a reliable basis for plague prevention and control, we analyzed the habitat characteristics of Himalayan marmots, developed Himalayan marmot information collection system V3.0 based on global navigation satellite system (GNSS), remote sensing, and geographic information system (“3S”) technology, and drew a predictive spatial distribution map of Himalayan marmots in Qinghai Province. Field survey data of 352 marmot plague sites in Qinghai Province were collected in 2014, and the data from 80 sample sites were included. The Himalayan marmot habitat characteristics were analyzed based on “3S” technology using five environment variables (elevation, slope, aspect, vegetation cover, and grass type) and the geographical coordinates. Himalayan marmot information collection system V3.0, which has been approved by the National Copyright Administration of the People’s Republic of China (No.00764743), was used to draw a predictive spatial distribution map of Himalayan marmots in Qinghai province. Moreover, from 2015 to 2017, positioning data of the plague-foci and plague-free areas in Qinghai Province were collected using GNSS receptor for field validations to verify the accuracy of the marmot predictive spatial distribution map. Elevation, slope, vegetation cover, and grassland type were identified as important environmental factors that determine the spatial distribution of Himalayan marmots. The suitable range of environmental features was 3400–4600 m elevation, 5°–20° slope, 0.60–1.00 vegetation cover, and alpine meadows. The Himalayan marmot predictive spatial distribution map in Qinghai Province based on “3S” technology and marmot information collection system V3.0 had a spatial resolution of 30 m. Field validation in areas of Qinghai Province revealed a prediction accuracy and mean absolute error of 0.8669 and 0.1331, respectively, which indicated excellent prediction accuracy. This study greatly improved the work efficiency of plague surveillance and effectively reduced the work intensity of researchers. Application of “3S” technology and marmot information collection system V3.0 has improved the data collection efficiency, provided new technical means for plague investigation and research, and provided a reference for development of plague surveillance programs. The research results will play a positive role in promoting the improvement and perfection of plague prevention and control strategies in Qinghai province and even in China.

## Introduction

Plague is a typical natural-focal disease and is a virulent infectious disease caused by *Yersinia pestis* that is characterized by rapid onset, strong infectivity, and high mortality^1^. Historically, there have been three worldwide pandemics of human plague throughout Asia, Europe, America, and most of Africa, which were highly disastrous to the global economy and human lives^2^. Recently, plague epidemics have shown an active trend worldwide, and the World Health Organization (WHO) has listed plague as a major resurging infectious disease^3^. Disease prevention and control in China have been developing, joint prevention and control mechanisms for infectious diseases have been improving, and the prevention and control of plague epidemics and emergency response capabilities have greatly improved. However, the natural plague foci of China are large, the geographical landscape within the natural foci is complex, the hosts are diverse, and population movement is becoming more frequent with the development of tourism. Therefore, plague prevention and control work should not be relaxed in China.

The natural Himalayan marmot plague focus on the Qinghai Tibet Plateau (QTP) is the most active and widely distributed of the 12 natural plague foci in China. More than 30 plague-stricken counties have been found in Qinghai province, with an epidemic area of nearly 200,000 km. These areas have serious animal plague epidemics and increased risk of human plague, which seriously endangers local socioeconomic and production activities^4^. The Himalayan marmot, a large terrestrial rodent unique to the QTP, is the most important host animal of Himalayan marmot plague in Qinghai province and is the main source of human plague infection, accounting for 73.17% of human plague infection^5,6^.

Within a specific geographic area, the spatial distribution of the host animals is determined by topography, elevation, vegetation, and other geographic landscape elements. The distribution of host animals is in turn closely related to the activity of the local plague epidemic and has an important influence on the spatiotemporal dynamic distribution of plague natural foci^7–15^. Animal-based plague surveillance is the main plague prevention and control measure in China^16^, and it is the main strategy used in Qinghai province to improve the effectiveness of plague surveillance in marmots, detect the marmot plague epidemic in a timely and accurate manner, correctly judge the marmot plague epidemic trend, deploy measures to prevent and control plague timely, and strictly prevent the plague from spreading to humans. However, given the large geographical coordinates range, diverse geographical landscape, and complex ecosystem in Qinghai province, it is time-consuming and inefficient to conduct on-site surveys of plague host animals on a large scale.

The “3S” technology, which includes global navigation satellite system (GNSS), remote sensing (RS), and geographic information system (GIS), has the advantages of wide coverage (such as high spatial, spectral and temporal resolution), high efficiency, time saving, labor saving, and scientific objectivity, and thus is widely applied in a wide range of fields including ecological environment data monitoring, air quality detection, monitoring of the spatial distribution of species, infectious disease monitoring, and health-related topics research^17–23^. “3S” technology also has been used in plague surveillance, risk assessment, and prevention and control^24–27^. However, due to the particularity of different plague foci, surveillance system based on “3S” technology for one focus is not applicable to plague monitoring and control in all other plague foci. In this study, data obtained by “3S” technology and survey data of Himalayan marmot activity sample sites were used for preliminary quantitative statistical analysis. These data were then included in marmot information collection system V3.0 to draw a predictive spatial distribution map of Himalayan marmot in the natural plague foci in Qinghai province. The marmot plague distribution was predicted to provide guidance for plague surveillance in Qinghai province and a reliable basis for plague prevention and control.

## Materials and methods

The specifications of all the data were list in the Supplementary Table S1.

### Data sources

Marmot information collection system V3.0 was developed by the Qinghai Provincial Basic Geographic Information Center.

Marmot plague survey data were provided by the Qinghai Institute for Endemic Diseases Prevention and Control. The spatial distribution of marmots in 352 sample sites in Yushu, Guoluo, Ulan, Jianza, Xinghai, and Qilian were collected in 2014 by field survey using GNSS (U.S. navigation star global positioning system, USA).

Marmot habitat data were obtained using five environmental variables, including elevation, slope, aspect, vegetation cover, and grassland type. Elevation, slope, and aspect were obtained from the Qinghai Provincial Geographic Information Center with a resolution of 30 m; vegetation cover data were estimated by the image element decomposition model using the RS image data from Landsat Operational Land imager (OLI) of Landsat Data Continuity Mission (LandSat8; NASA, USA) of 2014; and grassland type data were determined from the second national RS grassland survey of China (the first national grassland survey was carried out during 1984–1997, the second one was performed during 2017–2018, and the third one was conducted in 2019). The longitude and latitude of the sample sites were collected by GNSS receptor.

Field validation verification data were provided by the Qinghai Institute for Endemic Diseases Prevention and Control. To further verify the accuracy of the predicted spatial distribution map of marmot, the spatial positioning data of plague foci and plague-free areas in Qinghai province were collected using GNSS receptor from 2015 to 2017. This included 352 sample sites in Xining, Haidong, Haixi, Hainan, and parts of Haibei.

### Data processing

The ArcGIS 10.2 software (Environmental Systems Research Institute, Inc., USA) was used to incorporate the marmot activity sample site data into the CGCS2000 (China Geodetic Coordinate System 2000, China) coordinate system based on the geographical coordinates data collected by GNSS receptor. Sample sites with a distance of less than 3 km were excluded so that the sample sites could be evenly distributed in the areas with marmots. A total of 80 sample sites were obtained after excluding the redundant sample sites (Table S2).

### Vegetation cover data

The Optimized Soil-Adjusted Vegetation Index (OSAVI) correlated well with the vegetation cover in Qinghai province^28–30^. OSAVI can reflect the growth of plants, and is calculated as following: 1.16 × (*R*_*nir*_ − *R*_*red*_)/(*R*_*nir*_ + *R*_*red*_ + 1.16). Where *R*_*nir*_ is the reflectance value of remotely sensed image in the near infrared band, *R*_*red*_ is the reflectance value of the remotely sensed image in the infra-red band. OSAVI was used to estimate the vegetation cover in August in Qinghai province. The vegetation cover was estimated using the following foumula: (OSAVI − OSAVI_*soil*_)/(OSAVI_*veg*_ − OSAVI_*soil*_)^28–30^. Where OSAVI_*soil*_ is the OSAVI value of the fully bare-covered area, OSAVI_*veg*_ is the OSAVI value of the fully vegetation-covered area. The values of vegetation cover are between 0 and 1, with 0 representing non-vegetation covered areas such as water bodies, snow and ice, values close to 0 representing soil or rocks, and values closer to 1 representing higher vegetation coverage and more vegetation.

### Grassland type

Grassland type data was obtained from the second national RS grassland survey of China. These data subdivided the grassland types in Qinghai province into 9 major grassland classes, 10 grassland subclasses, 28 grassland groups, and 173 grassland types.

### Elevation data

The selection of marmot habitat is largely influenced by geographical factors, and the suitable elevation for Himalayan marmots is 2200–5200 m^31^. The elevation data of the basic sample sites were extracted based on the 1: 50,000 digital elevation model (DEM) of Qinghai province (Fig. S1A) and the frequency of the extracted elevation values was analyzed to infer the possible spatial distribution areas of Himalayan marmots.

### Slope and aspect data

Both slope and aspect values were calculated from the elevation data. The DEM data of the Qinghai province (Fig. S1A) was used to perform slope and aspect analysis in ArcGIS to derive a map of slope merging with aspect (Fig. S1B). The frequency of the extracted slope and aspect values was analyzed to infer the possible spatial distribution areas of Himalayan marmots.

### Reclassification of the data

The purpose of reclassification was to overcome the limitations of spectral information such as ‘different objects with the same spectra characteristics’ and ‘the same objects with different spectra characteristics’ in RS images, and to calibrate all data. The rules of data reclassification were different based on the data characteristics, the purpose of data use, and the convenience of late calculation. The reclassification of the data was specifically operated in the GIS software. Thus, some of the data were specifically reclassified using the GIS software of geographic information specialty.

### Construction of marmot information collection system V3.0

**Figure 1:**
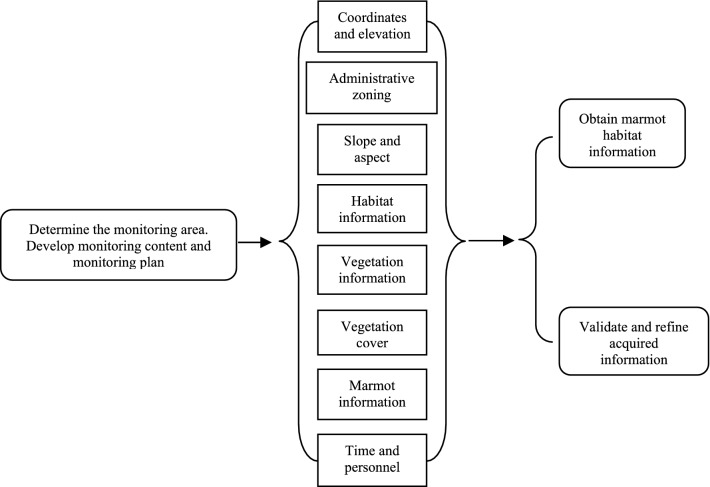
Workflow of Himalayan marmot surveillance.

The main purpose of the fieldwork was to monitor the spatial and geographic information of an area. With the main objective of surveillance of the actual marmot spatial distribution, natural elements such as vegetation and soil and the presence or absence of marmots provide a basis for extrapolating marmot distribution. The workflow for the Himalayan marmot surveillance is listed as Fig. 1.

**Figure 2:**
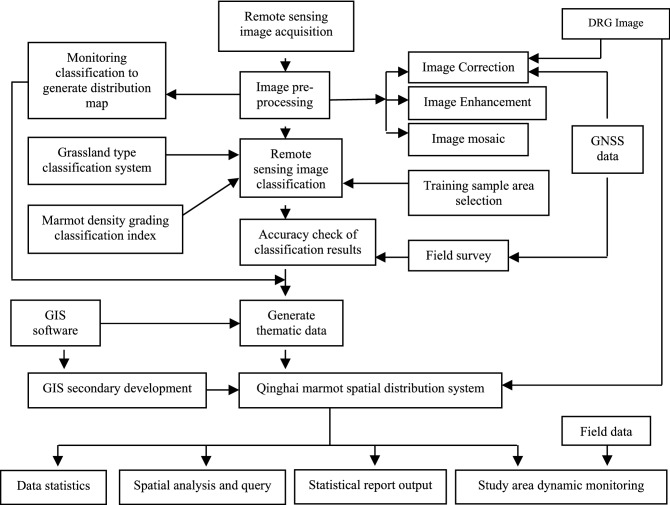
Schematic diagram of marmot information collection system V3.0 Data verification by field validation.

Marmot information collection system V3.0 was designed based on the widely used Android 7.0 system (Google, USA. Version number: NRD90M.T715CZCS2CTK1) and was established using the development platform described in Data Sources section. The schematic diagram is shown in Fig. 2.

The field validation data from 2015 to 2017 collected by the marmot information collection system were validated using the mean absolute error (MAE) model to ensure the accuracy of the predicted spatial distribution of Himalayan marmots.

${\text{MAE}} = \frac{1}{{\text{n}}}\sum\nolimits_{{{\text{i}} = 1}}^{{\text{n}}} {\left| {{\text{y}}_{{\text{i}}} - {\overline{\text{y}}}_{{\text{i}}} } \right|}$,where ${\mathrm{y}}_{\mathrm{i}}$ is the predicted value from field validation data, ${\overline{\mathrm{y}} }_{\mathrm{i}}$ is the average of the predicted probability of Himalayan marmot distribution from all field validation data, and n is the number of sample sites with Himalayan marmot activity or burrow distribution found in the field validation. MAE is the average of the absolute errors.

Accuracy of predicted Himalayan marmot spatial distribution = 1 − MAE.

## Results

### Active marmot sample sites

As shown in supplementary Fig. S1A, the 80 sample sites were distributed in Haidong, Hainan, Guoluo, and Yushu. Of these, 30 were found to be inhabited by Himalayan marmots and 12 had a historical outbreak of plague among marmots. The other sample sites had marmot burrows, and therefore can be considered reliable distribution sites of Himalayan marmots.

### Vegetation cover extraction and analysis

**Figure 3:**
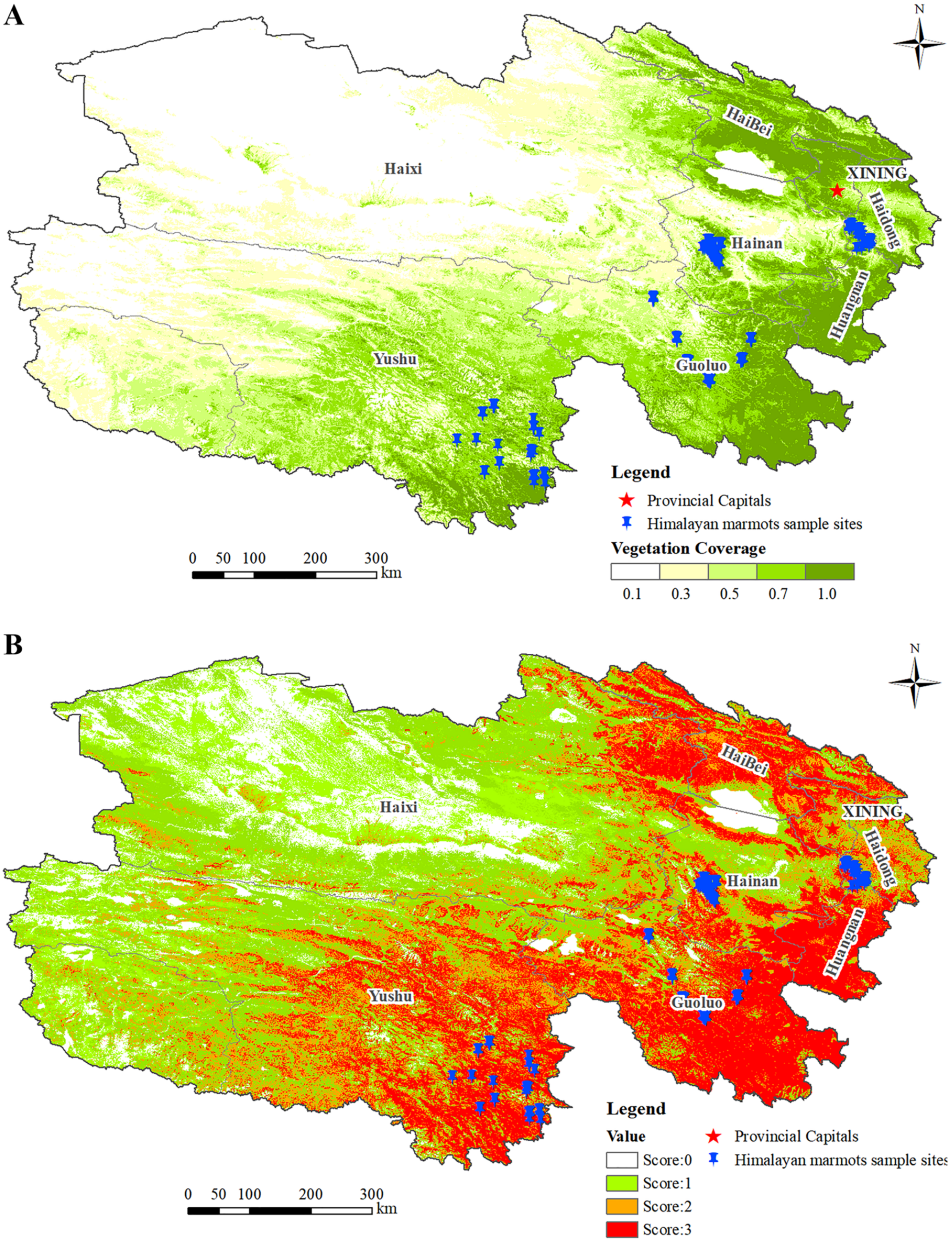
Vegetation cover in Qinghai Province (**A**); Reclassification of vegetation cover (**B**).

The vegetation cover of the sites extracted from the field survey data was shown in Fig. 3A. Analysis of the frequencies of the extracted vegetation cover values revealed that the maximum value of vegetation cover at the sample sites was 0.9999, the minimum value was 0.1268, the mean value was 0.6507, and the standard deviation was 0.2558. There are 8, 28, and 44 sample sites with vegetation cover between 0.12 and 0.25, 0.25 and 0.60, and 0.60 and 1.0, respectively, accounting for 10.00%, 35.00%, and 55.00% (Table 1). No sample sites were detected with the vegetation cover value lower than 0.12 or higher than 1.0.

**Table 1:** Analysis of marmot sample sites under different vegetation cover classifications.

Type	Vegetation cover classification	Number of sample sites	Proportion (%)
0	< 0.12	0	0.00
1	0.12–0.25	8	10.00
2	0.25–0.60	28	35.00
3	0.60–1.00	44	55.00
4	> 1.0	0	0.00

The active marmot sample sites were mainly distributed in areas with vegetation cover of 0.60–1.00, which were classified as one category and assigned a value of 3. Similarly, areas with vegetation cover of 0.25–0.60 were assigned a value of 2, areas with vegetation cover of 0.12–0.25 were assigned a value of 1, and the rest were assigned a value of 0. Reclassification of vegetation cover is shown in Fig. 3B.

### Marmot distribution and grassland types

**Figure 4:**
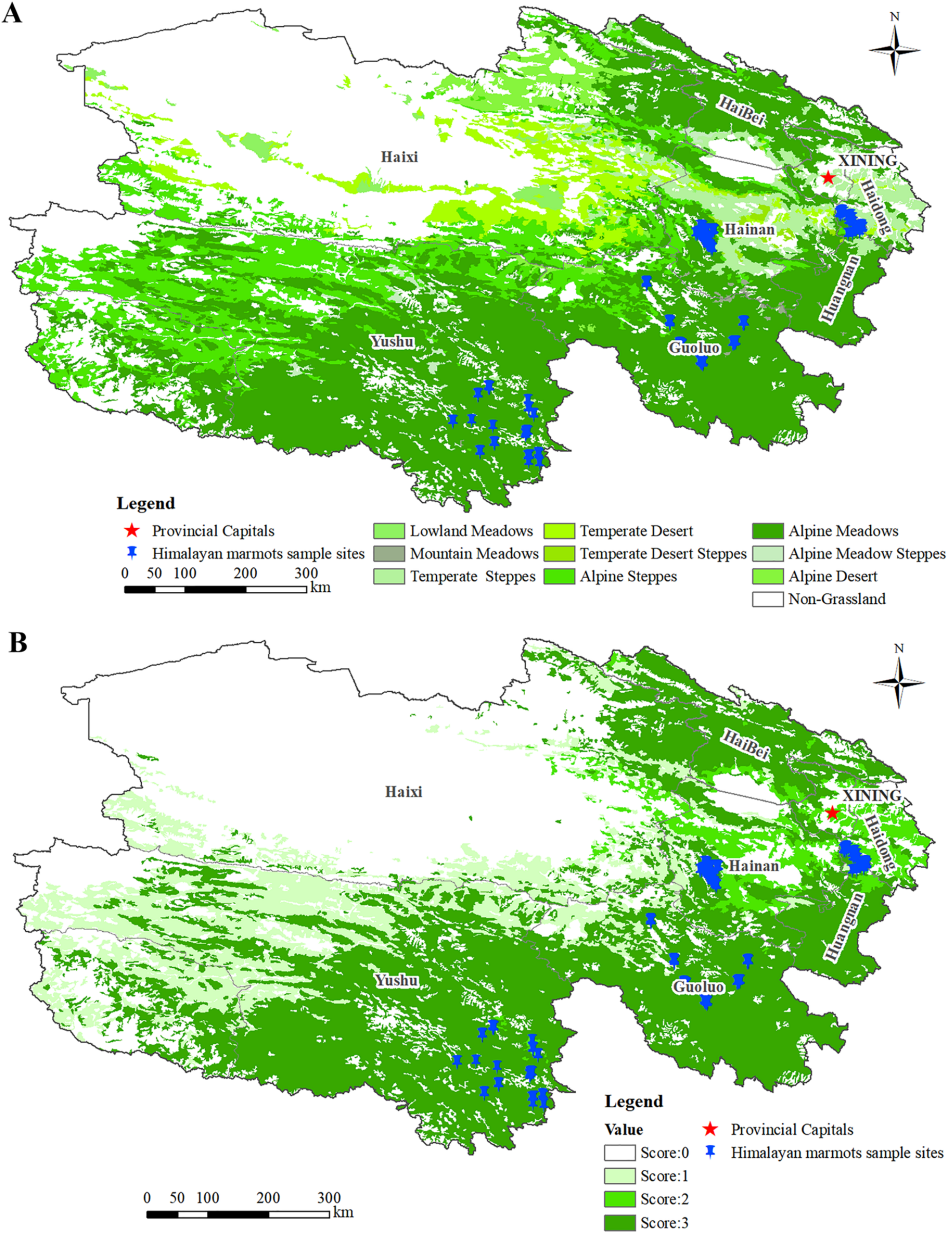
Province-wide grassland types (**A**); Reclassification of grassland types (**B**).

Marmots were more active in sunny meadow grassland areas with good vegetation, lush growth of grasses, more dominant plants, and abundant food. In contrast, there were almost no marmots distributed in the alpine scrub forest, coniferous forest, and alpine snow/ice covered areas where vegetation growth was poor, vegetation cover was low, and food was scarce (data not shown). The data based on analysis of the second national RS grassland survey revealed that there are 58 alpine meadows, 18 temperate grasslands, and 4 alpine grasslands, accounting for 72.50%, 22.50%, and 5.00% of the total of 80 sample sites, respectively (Fig. 4A, Table 2).

**Table 2:** Statistics on the number of marmot distribution sites in different grassland types.

Type	Grassland type	Number of sample sites	Proportion (%)
1	Lowland meadow	0	0.00
2	Montane meadow	0	0.00
3	Temperate grassland	18	22.50
4	Temperate desert steppe	0	0.00
5	Alpine grassland	4	5.00
6	Alpine meadow	58	72.50
7	Alpine meadow grassland	0	0.00
8	Non-grassland	0	0.00

The number of Himalayan marmots significantly varied depending on grassland type, with the highest density of marmot distribution sites in alpine meadows, a few marmot distribution sites in temperate grassland and alpine grassland, and almost no marmot distribution sites in other types of grasslands (Table 2). The highest distribution of marmots was observed in alpine meadows, which were assigned a value of 3, followed by temperate grasslands, which were assigned a value of 2, and alpine grasslands, which were assigned a value of 1. The other types of grassland had no marmots and were assigned a value of 0. Reclassification of grassland types based on marmot distribution is shown in Fig. 4B.

### Marmot distribution and elevation

Frequency statistics of the extracted elevation values for the sample sites revealed a maximum elevation of 4650 m, minimum elevation of 2035 m, and mean elevation of 3600 m with standard deviation of 643 m. The number and proportion of sample sites in different elevation classifications are listed in Table 3, showing that a total of 61, 18, and 1 sample sites were located in the elevation of 3400–4600 m, 2000–3400 m, and 4600–5200 m, respectively, accounting for 76.25%, 22.50%, and 1.25%.

**Table 3:** Statistics on the number of sample sites classified by different elevations.

Type	Elevation classification	Number of sample sites	Proportion (%)
0	< 2000	0	0.00
1	2000–3400	18	22.50
2	3400–4600	61	76.25
3	4600–5200	1	1.25
4	> 5200	0	0.00

**Figure 5:**
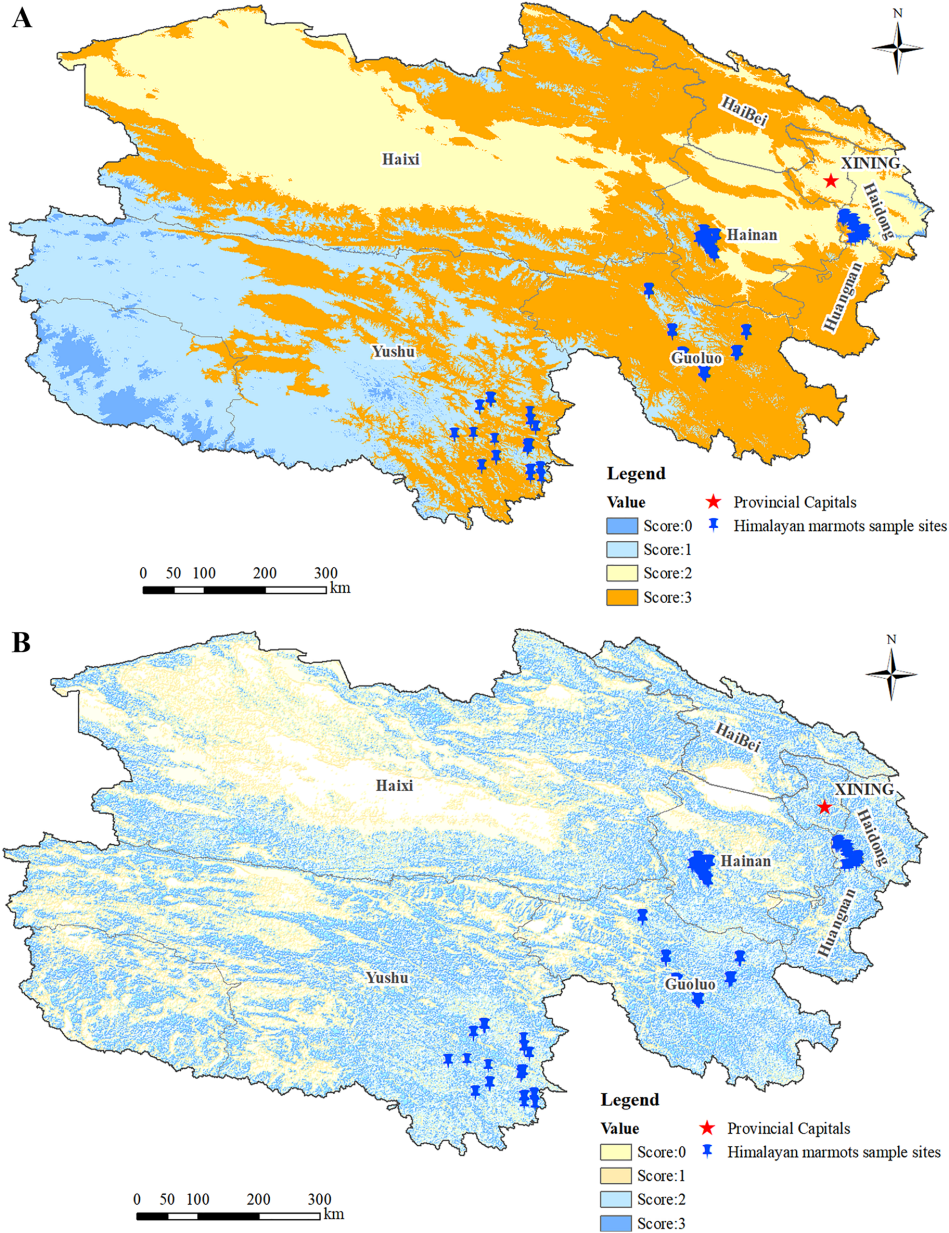
Reclassification based on elevation (**A**); Reclassification based on slope and aspect (**B**).

Marmots were mostly distributed between 3400 and 4600 m, which was thus classified as one category and assigned a value of 3. Similarly, elevation of 2000–3400 m was assigned a value of 2, elevation of 4600–5200 m was assigned a value of 1, and other elevations were assigned a value of 0. The reclassified DEM of the Qinghai province using these values is shown in Fig. 5A.

### Marmot distribution and slope

Slope analysis of the sample sites conducted in ArcGIS using the 1:50,000 DEM of Qinghai province revealed a maximum slope of 30.45°, minimum slope of 0.41°, and mean slope of 11.64° with standard deviation of 8.97°. The proportions of the number of sites found in different slope classifications are shown in Table 4, showing that there are 43, 25, and 12 sample sites with slopes between 5 and 20, 0.5 and 5, and 20 and 31, respectively, accounting for 53.75%, 31.25%, and 15.00%.

**Table 4:** Statistics on the number of marmot distribution sites classified by different slopes.

Type	Slope classification	Number of sample sites	Proportion (%)
0	< 0.4	0	0.00
1	0.4–5	25	31.25
2	5–20	43	53.75
3	20–31	12	15.00
4	> 31	0	0.00

There were no Himalayan marmot distribution sites in areas with slopes greater than 31°; most marmot distribution sites were found in areas with slopes of 5–20°, and a few were found in areas with slopes of 0–5° and 20–31°. The marmot distribution sites with slopes of 5–20° were classified into one category and assigned a value of 3. Similarly, sites with slopes of 20–31° were assigned a value of 2, sites with slopes of 0.4–5° were assigned a value of 1, and the rest were classified as one category and assigned a value of 0. The reclassified digital slope and aspect of Qinghai province using these values is shown in Fig. 5B.

### Mapping the spatial distribution of Himalayan marmots in Qinghai province

**Figure 6:**
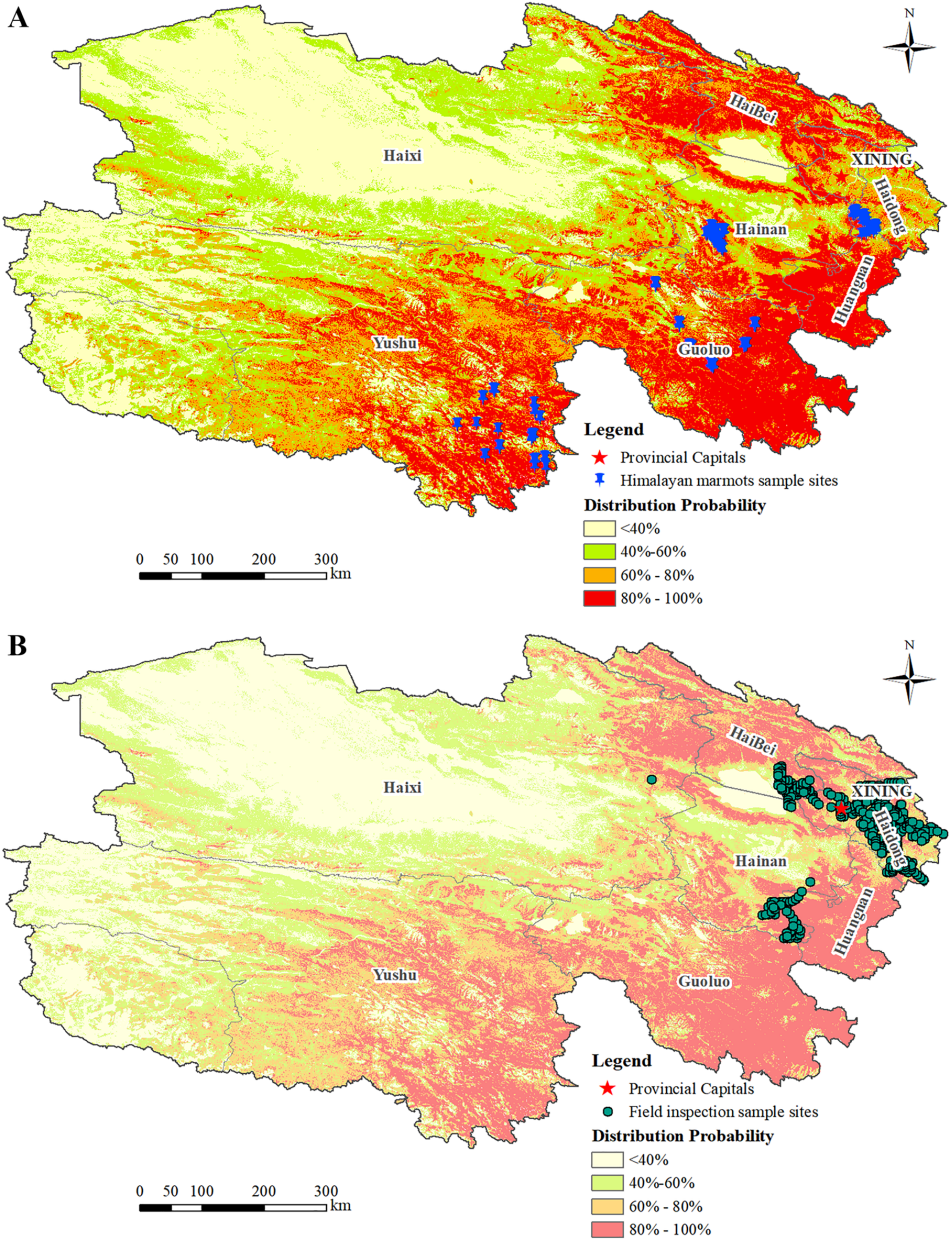
Predicted Himalayan marmot spatial distribution in Qinghai Province (**A**); Spatial distribution map of field visit survey sites in Qinghai Province from 2015 to 2017 to verify the prediction results (**B**).

As shown in Fig. 6A, the probability of marmot distribution in the < 40% area was the smallest, with an area of approximately 198,581.34 km^2^, and was mainly distributed in the desert and mountainous areas in the western part of Qinghai province. The area with 40–60% probability of marmot distribution was approximately 199,695.86 km^2^, and was mainly distributed in the intersection of desert and grassland. The area with 60–80% probability of marmot distribution was approximately 119,676.47 km^2^, and was mainly distributed in the temperate grassland. The area with 80–100% probability of marmot distribution was approximately 174,654.97 km^2^ and accounted for approximately 25% of the total area of Qinghai province; this area was mainly located in the southeastern part of Qinghai province with gentle terrain, abundant water and grass, and sufficient sunshine.

Himalayan marmots in Qinghai were mainly distributed in the eastern and southern parts of Qinghai and the northern parts of Qilian, Gangcha, and Tianjun, which mainly have the alpine meadow grassland type and a vegetation cover range of 0.60–1.00 (Fig. 6B). The central and northwestern parts of Qinghai had fewer marmot distribution sites or were not suitable for marmots. The density of marmot distribution in Qinghai province showed a gradual decrease from southeast to northwest.

### Main functions of marmot information collection system V3.0

We developed an application, namely marmot information collection system V3.0, which has been approved by the National Copyright Administration of the People’s Republic of China (No.00764743), to collect marmot information. The basic functions of the software mainly include map control (zooming and roaming), layer control, overlay of raster and vector layers, distance measurement, data spatial visualization, information query, spatial analysis and statistics, and thematic map output. By including information related to the Himalayan marmot habitat of the natural plague foci in Qinghai province, this system can be used for management and analysis of plague surveillance and survey data; storage of spatial data, attribute data, and their associated image data using files in the application database and exported according to different task layers and different storage methods; historical epidemic data importation, route planning, real-time positioning navigation, and playback function tracking.

### Data verification by field validation

Results of field validations in the plague-infected and plague-free counties of Haiyan, Tongde, Xunhua, Huzhu, Huanzhong, Ledu, Datong, and Qilian from 2015 to 2017 are shown in Fig. 6B. Among the 352 spatially independent sites surveyed in 2016–2017, 68 were determined to have Himalayan marmots or active burrows. The prediction accuracy results based on the error function revealed a prediction accuracy and mean absolute error of 0.8669 and 0.1331.

## Discussion

### Himalayan marmot habitat analysis

The environmental factors including temperature, vegetation and elevation are the key drivers for the wildlife in alpine ecosystems^32^. Specific landform attributes such as slope and elevation and vegetation cover affect the population and burrowing of rodents^33^. For example, rodent burrows in the Western Usambara Mountains in Tanzania were only found at an elevation of above 1600 m^33^. However, the Himalayan marmot seems to prefer to inhabit areas with low elevation and high land surface temperature^34^. In this study, the data showed that 76.25% of the Himalayan marmots were found in areas with elevation values of 3400–4600 m. The majority of marmots were found in areas with slopes of 5–20° and vegetation cover higher than 60%. Most marmots were found in alpine meadows, a few were found in temperate grasslands and alpine grasslands, and none were found in other grassland types.

Preliminary statistical analysis of vegetation cover, grass type, vegetation type, and Himalayan marmot distribution sample sites obtained using spatial geographic information technology revealed that the meadow grassland areas with lush grass growth, more dominant plants, and abundant food had more marmots. When the vegetation cover reached 0.60–1.00, the number of marmot distribution sample sites was the highest. Dense grass is an ideal habitat and provides concealment for Himalayan marmots, and the abundant plant types provide sufficient food for marmots. In contrast, no marmots were distributed in the alpine scrub, coniferous forest, and alpine snow/ice covered areas where vegetation growth was poor, vegetation cover was low, and food was relatively scarce. Moreover, 70.24% of Himalayan marmots were found in alpine meadows with a wide variety of plant species, including *Poaceae*, *Cyperaceae,* and grasses. This finding indicated that alpine meadows are more suitable for Himalayan marmots and have more advantageous habitat conditions compared with other grassland types. The elevation of alpine meadows is 3236–5126 m, and the vegetation is mainly meadows with simple vegetation structure, substantial vegetation cover and dense vegetation growth, and a wide variety of plants, rich food, soft grass, and good palatability. Therefore, alpine meadows provide good natural habitats and foraging sites for marmots.

Habitat selection of large rodents is influenced by a combination of vegetation cover availability, food availability, and population density^35^. Vegetation cover is an important parameter that describes vegetation communities and ecosystems and is closely related to vegetation quantity and productivity. The quality of habitat vegetation is an important factor that affects the spatial distribution of plateau rodents. Both feeding and concealment depend on vegetation, and the height and cover of edible plants and vegetation suitable for concealment determine the choice of vegetation type by marmots. Thus, vegetation cover becomes an important factor for habitat selection by marmots. Different grassland types determine different plant conditions, and selection of different vegetation conditions can increase the chances of survival and improve the reproductive success of marmots; therefore, grassland type is an important ecological factor in habitat selection by marmots. A study showed that the ecological factors affecting habitat selection of Himalayan marmots are mainly topography, anthropogenic disturbance, and vegetation^8^. Another study concluded that habitat selection by Himalayan marmots is closely related to elements such as topography, landform, temperature, precipitation, and vegetation^24^.

The functions of burrows’ physical parameters is to protect the Himalayan marmots from natural enemies and bad weather^36^. There is clearly influence of slope on habitat selection by marmots. When the slope is large, wind is strong, and burrows are not well hidden; this makes them difficult to defend against enemies, unsafe for survival, and not conducive to hibernation during winter. In addition, Himalayan marmots prefer to burrow on sunny aspect, because the temperature is suitable and the vegetation is lush, which is suitable for marmots to breed. Therefore, the number of marmot burrows gradually decreases with increasing slope and ubac. Although flat and low-lying areas with small slopes are good for marmots to create dens, rainwater will easily flow into the dens during summer rainfall, which will kill marmots. Therefore, a suitable slope and sunny aspect are also very important for habitat selection by marmots.

### Application of the predictive spatial distribution map of Himalayan marmots in Qinghai province

Plague surveillance is the main measure used for plague prevention and control in China. Although we have made many improvements in plague surveillance, the traditional method of dragnet surveillance still consumes a lot of human and material resources, is inefficient. The pasture area of Qinghai province is approximately 380,000 km^2^, and the identified natural plague focus is approximately 180,000 km^2^; therefore, there is still 200,000 km^2^ of pasture where the distribution of Himalayan marmots and plague have not been identified. Currently, RS technology is widely used in the fields of mapping and ecological surveillance^18,19,21,22,37^.

Applications of RS technology in areas such as malaria, dengue, schistosomiasis and plague have been previously reported^27,37^. Using GIS combined with remotely sensed data, Proches Hieronimo et al. found that the presence of small mammals was positively influenced by elevation, whereas the presence of fleas was clearly influenced by land management features, and thus these observations have positive implications for plague surveillance^27^. In this study, RS technology combined with field validations were used to determine the distribution and areas of different types of grasslands in Qinghai province, and the average density of Himalayan marmot distribution in different types of grasslands. The high-, low-, and very low-density areas of Himalayan marmot distribution were identified. The soil map, vegetation map, administrative map, and marmot density statistics were merged to form the spatial data and attribute data basis for the information system to map the distribution of Himalayan marmot and determine the area of Himalayan marmot distribution. Generally speaking, the occurrence of human plague epidemic is closely related to the local animal plague epidemic^2^. However, a large part of the high-density distribution of Himalayan marmots is located in uninhabited areas and the areas are generally sparsely populated, which also indicates that we should reasonably allocate plague prevention and control resources to areas where human plague is most likely to occur to prevent the occurrence of human plague epidemics.

### Field validation for verification

Through field validation and information from local farmers and herdsmen, we confirmed that Himalayan marmots inhabited 68 sample sites in Tongde, Zeku, Guinan, Xunhua, Haiyan, Ulan, Qilian, Hualong, and Huzhu counties. Among them, Tongde, Zeku, Guinan, Xunhua, Haiyan, Ulan, and Qilian counties have all historically experienced marmot plague outbreaks and can be considered as reliable natural plague foci^38^. The data from this field validation are consistent with the previous survey data and the epidemic history of the counties in Qinghai province^39^.

MAE can better reflect the actual number of errors in prediction values; the smaller the MAE value, the higher the prediction accuracy. The MAE derived from the field validation data was 0.1331 and the prediction accuracy was 0.8669. The accuracy of the predicted Himalayan marmot spatial distribution reached 87%, which indicated that the predicted probability map of the Himalayan marmot spatial distribution can better predict the potential marmot distribution.

The predicted spatial distribution map of Himalayan marmot in Qinghai province was then compared with environmental information such as elevation, vegetation, grass type, slope, and aspect of 352 field survey sites. The obtained RS data showed that the prediction results were excellent, and the predicted spatial distribution map of Himalayan marmot in Qinghai province was drawn with high accuracy. The prediction map visually reflects the different density distribution of Himalayan marmots; this allows us to optimize the settings and reasonable spatial layout of animal plague surveillance sites and improve surveillance efficiency.

### Application of marmot information collection system V3.0

Marmot information collection system V3.0 was developed based on the “3S” technology standardizing the collection of surveillance data, and makes the management and analysis of information more convenient and faster. This study revolutionized the traditional method of considering plague-stricken counties as the plague foci, and effectively reduces the work intensity of operators and improves the data collection efficiency. In 2016 and 2017, we applied this system to the animal plague surveillance tasks in the plague-stricken counties of Haidong, Hainan, and Haibei in Qinghai province, and standardized the collection of provincial geographic location data of animal plague surveillance (data not shown). In 2018, we also applied this system in Wulan County, which frequently experiences plague, and achieved a good application effect (data not shown).

In the next step, we will expand the pilot areas (mainly national and provincial plague surveillance sites), collect surveillance data from each surveillance site, continuously optimize and update the system, improve the efficiency of data analysis and utilization, detect the plague epidemic in marmot in a timely and accurate manner, correctly determine the epidemic trend of plague in marmots, and attempt to strictly prevent the plague from spreading to humans. We plan to use a new model of drone surveillance to create a multidimensional, three-dimensional, real-time big data plague surveillance information reporting system to enhance early plague warnings and prediction in Qinghai province and even in the country, which will be of positive practical significance to serve and guarantee the Belt and Road Initiative. These approaches are expected to provide new technical means for plague investigation and research, and to provide references for setting up plague surveillance programs and prediction for the natural Himalayan marmot plague focus in Qinghai province and the QTP.

## Conclusions

In this study, the ‘3S’ technology combined with the marmot information collection system V3.0 were used to analyze the habitat characteristics of Himalayan marmot, calculate the potential distribution of marmot, and predict the plague risk in the Himalayan marmot plague focus of Qinghai province. This study greatly improved the work efficiency of plague surveillance and effectively reduced the work intensity of researchers. Application of “3S” technology and marmot information collection system V3.0 has improved the data collection efficiency, provided new technical means for plague investigation and research, and provided a reference for development of plague surveillance programs. These results will play a positive role in promoting the improvement and perfection of plague prevention and control strategies in Qinghai province and even in China.

## Supplementary Information

Supplementary Information.

## Data Availability

The original data presented in the study are included in the article/supplementary materials, further inquiries can be directed to the corresponding authors.
