# Supplementary material for: Analysis of Himalayan marmot distribution and plague risk in Qinghai province of China using the “3S” technology
Source: Sci Rep. 2023 Feb 2;13:1924. doi: 10.1038/s41598-023-28414-5 (PMC9894924; doi:10.1038/s41598-023-28414-5)
Supplement: Supplementary file 1 — Supplementary Information. [file 41598_2023_28414_MOESM1_ESM.doc]

**Supplementary Table S1. The specifications of the** **data**

| **Data** | **Source** | **Dates** | **Supplier** | **Coordinate system** | **Satellite image** |
| --- | --- | --- | --- | --- | --- |
| 352 sample sites | Field survey using GNSS | 2014 | Qinghai Institute for Endemic Diseases Prevention and Control | CGCS2000 | / |
| Marmot information collection system V3.0 | Designed and developed based on Android 7.0 system | 2015 | Qinghai Institute for Endemic Diseases Prevention and Control; Qinghai Provincial Basic Geographic Information Center | / | / |
| Geographical coordinates | ArcGIS 10.2; GNSS | 2014 | Qinghai Institute for Endemic Diseases Prevention and Control; Qinghai Provincial Basic Geographic Information Center | CGCS2000 | / |
| Elevation | ArcGIS 10.2; Digital elevation model (DEM) | 2014 | Qinghai Provincial Basic Geographic Information Center | CGCS2000 | / |
| Vegetation cover | RS image data；Landsat Operational Land imager (OLI) ;  ArcGIS 10.2; | 2014 | Qinghai Institute for Endemic Diseases Prevention and Control; Qinghai Provincial Basic Geographic Information Center | CGCS2000 | LandSat8; NASA |
| Grassland type | The second national RS grassland survey；ArcGIS 10.2 | 2017 | Qinghai Institute for Endemic Diseases Prevention and ControlQinghai Provincial Basic Geographic Information Center | CGCS2000 | / |
| Slope | The DEM data  ArcGIS 10.2 | 2014 | Qinghai Provincial Basic Geographic Information Center | CGCS2000 | / |
| Aspect | Field survey；ArcGIS 10.2 | 2014 | Qinghai Institute for Endemic Diseases Prevention and Control | / | / |
| Field validation | Field survey using GNSS | 2015-2017 | Qinghai Institute for Endemic Diseases Prevention and Control | CGCS2000 | / |

**Supplementary Table S2. Characteristics of the 80 analyzed sample site**s

| **Site No.** | **Longitude** | **Latitude** | **Elevation** | **Vegetation cover** | **Grassland type** | **Slope** | **Aspect** |
| --- | --- | --- | --- | --- | --- | --- | --- |
| Sample site 1 | 99.8303 | 35.8211 | 3,648.50 | 0.48 | Alpine grassland | 1.81 | Sunny |
| Sample site 2 | 99.8528 | 35.8373 | 3,976.66 | 0.21 | Alpine grassland | 20.01 | Sunny |
| Sample site 3 | 99.7471 | 35.8486 | 3,699.62 | 0.43 | Alpine meadow | 0.99 | Sunny |
| Sample site 4 | 99.7269 | 35.8167 | 3,654.06 | 0.59 | Alpine meadow | 2.73 | Semi-sunny |
| Sample site 5 | 99.7169 | 35.7959 | 3,614.74 | 0.35 | Alpine meadow | 0.76 | Sunny |
| Sample site 6 | 99.6441 | 35.9004 | 3,905.15 | 0.58 | Alpine meadow | 5.58 | Sunny |
| Sample site 7 | 99.6740 | 35.8555 | 3,771.73 | 0.58 | Alpine meadow | 1.88 | Semi-sunny |
| Sample site 8 | 99.6432 | 35.8146 | 3,730.82 | 0.13 | Alpine meadow | 8.94 | Sunny |
| Sample site 9 | 99.5784 | 35.7857 | 3,722.22 | 0.47 | Alpine grassland | 8.81 | Shady |
| Sample site 10 | 99.6152 | 35.7893 | 3,771.79 | 0.49 | Alpine meadow | 13.27 | Shady |
| Sample site 11 | 99.6568 | 35.7927 | 3,734.13 | 0.60 | Alpine meadow | 18.51 | Semi-shady |
| Sample site 12 | 99.6853 | 35.7753 | 3,649.53 | 0.49 | Alpine meadow | 12.72 | Semi-shady |
| Sample site 13 | 99.6913 | 35.7412 | 3,660.32 | 0.24 | Alpine meadow | 2.05 | Semi-sunny |
| Sample site 14 | 99.7066 | 35.7167 | 3,648.47 | 0.25 | Alpine meadow | 4.66 | Semi-shady |
| Sample site 15 | 99.7883 | 35.7015 | 3,593.18 | 0.60 | Alpine meadow | 19.22 | Sunny |
| Sample site 16 | 99.7284 | 35.6908 | 3,589.04 | 0.79 | Alpine meadow | 5.46 | Semi-shady |
| Sample site 17 | 99.7436 | 35.6593 | 3,577.28 | 0.84 | Alpine meadow | 2.82 | Shady |
| Sample site 18 | 99.7628 | 35.6191 | 3,560.06 | 0.75 | Alpine meadow | 1.76 | Semi-shady |
| Sample site 19 | 99.7997 | 35.6043 | 3,450.80 | 0.64 | Alpine meadow | 4.23 | Shady |
| Sample site 20 | 99.8280 | 35.5519 | 3,291.25 | 0.61 | Temperate grassland | 28.30 | Sunny |
| Sample site 21 | 96.2650 | 32.9725 | 4,375.51 | 1.00 | Alpine meadow | 5.19 | Shady |
| Sample site 22 | 95.9809 | 32.9633 | 4,303.10 | 0.97 | Alpine meadow | 11.93 | Semi-sunny |
| Sample site 23 | 97.2587 | 32.4505 | 4,205.26 | 1.00 | Alpine meadow | 26.44 | Semi-shady |
| Sample site 24 | 97.2470 | 32.4811 | 4,215.98 | 0.80 | Alpine meadow | 12.11 | Semi-sunny |
| Sample site 25 | 96.5946 | 32.6345 | 4,062.85 | 0.77 | Alpine meadow | 30.46 | Sunny |
| Sample site 26 | 97.2652 | 32.3246 | 3,957.09 | 0.85 | Alpine meadow | 2.83 | Semi-shady |
| Sample site 27 | 96.3804 | 32.4989 | 3,949.48 | 0.50 | Alpine meadow | 29.75 | Sunny |
| Sample site 28 | 97.0969 | 33.2693 | 3,716.55 | 0.98 | Temperate grassland | 9.36 | Sunny |
| Sample site 29 | 97.1815 | 33.0610 | 3,611.12 | 0.41 | Alpine meadow | 29.87 | Sunny |
| Sample site 30 | 96.5740 | 32.8880 | 4,429.99 | 1.00 | Alpine meadow | 5.68 | Sunny |
| Sample site 31 | 102.0132 | 35.8799 | 2,287.86 | 0.17 | Temperate grassland | 16.38 | Semi-shady |
| Sample site 32 | 102.0450 | 35.8530 | 2,511.46 | 0.34 | Temperate grassland | 29.09 | Shady |
| Sample site 33 | 101.9078 | 35.7853 | 3,472.06 | 0.49 | Alpine meadow | 1.98 | Sunny |
| Sample site 34 | 101.8359 | 35.7751 | 3,403.64 | 0.81 | Alpine grassland | 6.66 | Sunny |
| Sample site 35 | 102.0545 | 35.8784 | 2,035.30 | 0.62 | Temperate grassland | 4.63 | Semi-shady |
| Sample site 36 | 101.8536 | 35.9741 | 2,895.93 | 0.24 | Alpine meadow | 16.77 | Semi-shady |
| Sample site 37 | 101.8737 | 35.9957 | 2,810.60 | 0.69 | Temperate grassland | 24.16 | Semi-shady |
| Sample site 38 | 101.9054 | 36.0184 | 2,512.18 | 0.51 | Temperate grassland | 18.92 | Semi-shady |
| Sample site 39 | 101.8743 | 36.0680 | 2,272.78 | 0.20 | Temperate grassland | 6.65 | Semi-shady |
| Sample site 40 | 101.8575 | 36.0585 | 2,556.07 | 0.56 | Temperate grassland | 15.84 | Semi-shady |
| Sample site 41 | 101.8444 | 36.0222 | 2,888.35 | 0.89 | Temperate grassland | 24.01 | Semi-shady |
| Sample site 42 | 101.7733 | 36.1011 | 2,833.25 | 0.64 | Temperate grassland | 16.94 | Semi-shady |
| Sample site 43 | 101.7098 | 36.0857 | 2,947.36 | 0.75 | Temperate grassland | 5.41 | Sunny |
| Sample site 44 | 101.7579 | 36.1345 | 2,228.25 | 0.45 | Temperate grassland | 15.82 | Semi-sunny |
| Sample site 45 | 102.0128 | 35.8216 | 3,159.38 | 0.81 | Non-grassland | 19.85 | Sunny |
| Sample site 46 | 101.9382 | 35.8378 | 2,994.24 | 0.85 | Alpine meadow | 16.39 | Sunny |
| Sample site 47 | 102.0364 | 35.9075 | 2,215.09 | 0.36 | Temperate grassland | 4.59 | Shady |
| Sample site 48 | 101.9146 | 35.9526 | 2,445.87 | 0.39 | Temperate grassland | 14.64 | Sunny |
| Sample site 49 | 101.7294 | 36.1060 | 2,587.04 | 0.78 | Temperate grassland | 28.06 | Shady |
| Sample site 50 | 96.3530 | 33.3625 | 4,424.55 | 0.91 | Alpine meadow | 8.26 | Semi-shady |
| Sample site 51 | 99.3415 | 34.1290 | 4,368.67 | 0.48 | Alpine meadow | 4.47 | Shady |
| Sample site 52 | 97.1124 | 32.4369 | 4,355.37 | 0.75 | Alpine meadow | 29.11 | Sunny |
| Sample site 53 | 99.1947 | 34.4655 | 4,328.55 | 0.42 | Alpine meadow | 5.22 | Shady |
| Sample site 54 | 99.3674 | 34.1058 | 4,378.97 | 0.59 | Alpine meadow | 19.86 | Sunny |
| Sample site 55 | 99.2024 | 34.4302 | 4,304.75 | 0.35 | Alpine meadow | 3.51 | Semi-shady |
| Sample site 56 | 99.3422 | 34.1096 | 4,436.06 | 0.78 | Alpine meadow | 14.16 | Shady |
| Sample site 57 | 97.1045 | 32.3424 | 4,650.10 | 1.00 | Alpine meadow | 14.11 | Semi-sunny |
| Sample site 58 | 99.2000 | 34.4487 | 4,304.82 | 0.46 | Alpine meadow | 3.08 | Semi-shady |
| Sample site 59 | 99.3742 | 34.1201 | 4,335.80 | 0.49 | Alpine meadow | 2.11 | Semi-shady |
| Sample site 60 | 99.3732 | 34.0901 | 4,288.27 | 0.16 | Alpine meadow | 8.56 | Sunny |
| Sample site 61 | 98.8643 | 35.0310 | 4,302.80 | 0.36 | Alpine meadow | 0.84 | Semi-shady |
| Sample site 62 | 98.8530 | 35.0604 | 4,278.98 | 0.59 | Alpine meadow | 1.33 | Semi-sunny |
| Sample site 63 | 97.0978 | 32.4249 | 4,239.90 | 1.00 | Alpine meadow | 18.56 | Semi-sunny |
| Sample site 64 | 100.1362 | 34.1339 | 4,107.09 | 0.99 | Alpine meadow | 17.47 | Semi-sunny |
| Sample site 65 | 100.1754 | 34.1520 | 4,079.29 | 0.81 | Alpine meadow | 5.97 | Sunny |
| Sample site 66 | 100.1544 | 34.1521 | 4,075.56 | 0.92 | Alpine meadow | 1.72 | Semi-sunny |
| Sample site 67 | 97.0560 | 32.7569 | 4,071.03 | 1.00 | Alpine meadow | 19.50 | Semi-sunny |
| Sample site 68 | 99.6493 | 33.8588 | 3,982.12 | 0.91 | Alpine meadow | 3.27 | Semi-sunny |
| Sample site 69 | 96.5266 | 33.4622 | 3,982.04 | 0.46 | Alpine meadow | 12.64 | Semi-shady |
| Sample site 70 | 99.6900 | 33.8244 | 3,993.73 | 0.61 | Alpine meadow | 18.35 | Semi-shady |
| Sample site 71 | 99.6754 | 33.8444 | 3,978.05 | 0.55 | Alpine meadow | 1.10 | Shady |
| Sample site 72 | 96.5145 | 33.4756 | 3,965.10 | 1.00 | Alpine meadow | 6.11 | Shady |
| Sample site 73 | 97.0735 | 32.7907 | 3,992.48 | 1.00 | Alpine meadow | 19.86 | Sunny |
| Sample site 74 | 97.0964 | 32.7966 | 4,339.00 | 0.96 | Alpine meadow | 28.44 | Shady |
| Sample site 75 | 97.0731 | 32.8055 | 3,947.26 | 0.99 | Alpine meadow | 3.36 | Shady |
| Sample site 76 | 97.1099 | 33.1560 | 3,777.29 | 0.98 | Alpine meadow | 11.79 | Semi-sunny |
| Sample site 77 | 100.2932 | 34.4552 | 3,824.79 | 0.90 | Alpine meadow | 18.69 | Shady |
| Sample site 78 | 100.2998 | 34.4429 | 3,772.21 | 0.84 | Alpine meadow | 6.52 | Sunny |
| Sample site 79 | 100.2801 | 34.4459 | 3,753.56 | 0.86 | Alpine meadow | 0.41 | Sunny |
| Sample site 80 | 97.1015 | 33.1503 | 3,636.90 | 1.00 | Temperate grassland | 7.54 | Sunny |


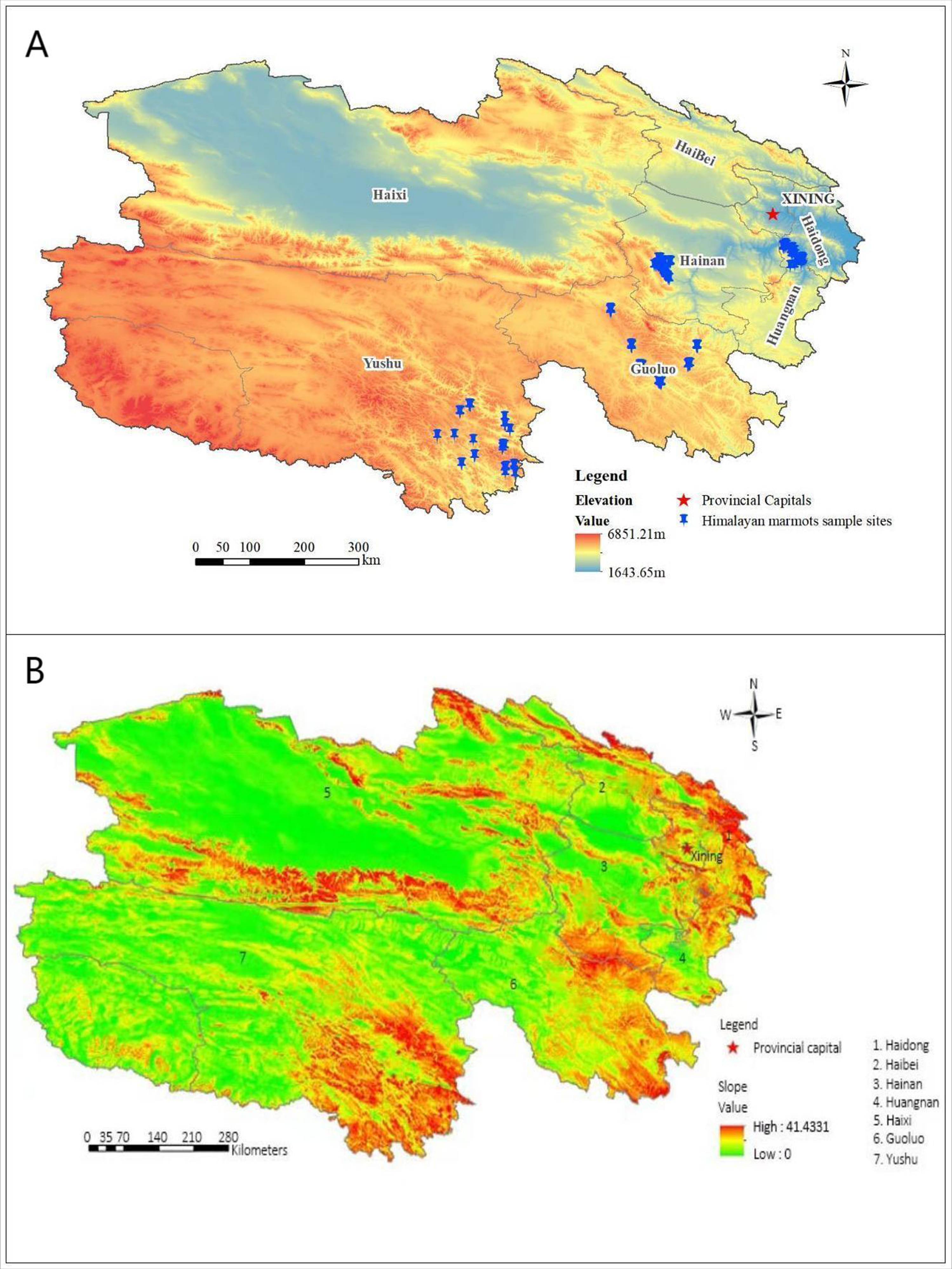


**Supplementary Figure S1. Province-wide 1:50,000 digital elevation model and Spatial distribution of sample sites (A); Province-wide slope analysis (B).**
